# The Importance of Using Exosome-Loaded miRNA for the Treatment of Spinal Cord Injury

**DOI:** 10.1007/s12035-022-03088-8

**Published:** 2022-10-24

**Authors:** Yunpeng Shen, Junying Cai

**Affiliations:** Department of Anesthesiology, The Second Affiliated Hospital of Nanchang University, Nanchang, 330006 Jiangxi China

**Keywords:** Exosomes, miRNA, Spinal cord injury, Treatment, Stem cells

## Abstract

Spinal cord injury (SCI) is a major traumatic disease of the central nervous system characterized by high rates of disability and mortality. Many studies have shown that SCI can be divided into the two stages of primary and secondary injury. Primary injury leads to pathophysiological changes, while consequential injury is even more fatal, including a series of harmful reactions that expand the scope and degree of SCI. Because the pathological process of SCI is highly complex, there is still no clear and effective clinical treatment strategy. Exosomes, membrane-bound extracellular vesicles (EVs) with a diameter of 30–200 nm, have emerged as an ideal vector to deliver therapeutic molecules. At the same time, increasing numbers of studies have shown that miRNAs play a momentous role in the process of SCI. In recent studies, researchers have adopted exosomes as carriers of miRNAs with potential therapeutic effects in SCI. In this review, we summarize relevant articles describing exosomes as miRNA carriers for SCI, after which we discuss further implications and perspectives of this novel treatment modality.

## Introduction

Acute spinal cord injury (SCI) is a severe traumatic event. On the basis of the World Health Organization (WHO) estimates, there are approximately 250,000–500,000 new cases of SCI in the world each year [1]. When a SCI occurs, the patient’s normal sensory, motor, or autonomic functions are greatly affected. SCI is primarily caused by spinal misalignment, which results in immediate neuronal death, vascular rupture, and blood spinal cord barrier (BSCB) damage. Furthermore, the wound microenvironment leads to further neurological damage and dysfunction, including neuronal apoptosis, inflammatory response, vascular changes, free radical accumulation, and glial cell activation, which will further prolong the acute phase of SCI.

SCI is a devastating neurological disorder that affects thousands of individuals each year [2]. However, because of its complex pathophysiological process, there are no clear and effective targeted therapies and functional recovery strategies for SCI. Consequently, the main research directions today mainly include (1) cell therapy, which uses the secretory function of stem cells to create a favorable environment for axonal regeneration and further induces differentiation into neurons to reconstruct neural connections across cells; (2) molecular intervention treatments, i.e., utilizing hormones and multiple cytokines to protect neural cells from secondary damage, promote axonal regeneration, and strengthen their conduction function; and (3) acupuncture and motor rehabilitation training treatments. The role of stem cells in SCI has been studied extensively, but many studies have shown that stem cells have many defects and that their therapeutic effects on SCI are most likely related to paracrine signals. Exosomes have attracted attention in recent years as an important mediator of cell-to-cell communication that are involved in many pathological processes [3].

Exosomes are small, single-membrane, secreted organelles with a diameter of ∼30 to ∼200 nm that have the same topology as the cell and are enriched in selected proteins, lipids, nucleic acids, and glycoconjugates [4]. Exosomes have functions in remodeling the extracellular matrix and delivering signal molecules to other cells. This intercellular vesicular trafficking plays an important role in various aspects of human health and disease. Based on these properties, exosomes have been developed as therapeutic agents in a variety of disease models. MicroRNAs (miRNAs), small non-coding RNA molecules that negatively regulate gene expression, can serve as diagnostic biomarkers and are emerging as novel therapeutic targets for central nervous system injury [5, 6]. The use of a combination of exosomes and miRNAs in SCI is increasingly recognized as a promising therapeutic strategy. Several studies to date have shown that miRNAs transported by exosomes from various cells have a significant protective effect in SCI [1]. For these reasons, scholars are now increasingly beginning to join this line of research to deeply investigate the positive effects of the combination of exosomes with miRNAs on SCI and the underlying mechanisms.

## Exosome Biogenesis

**Fig. 1:**
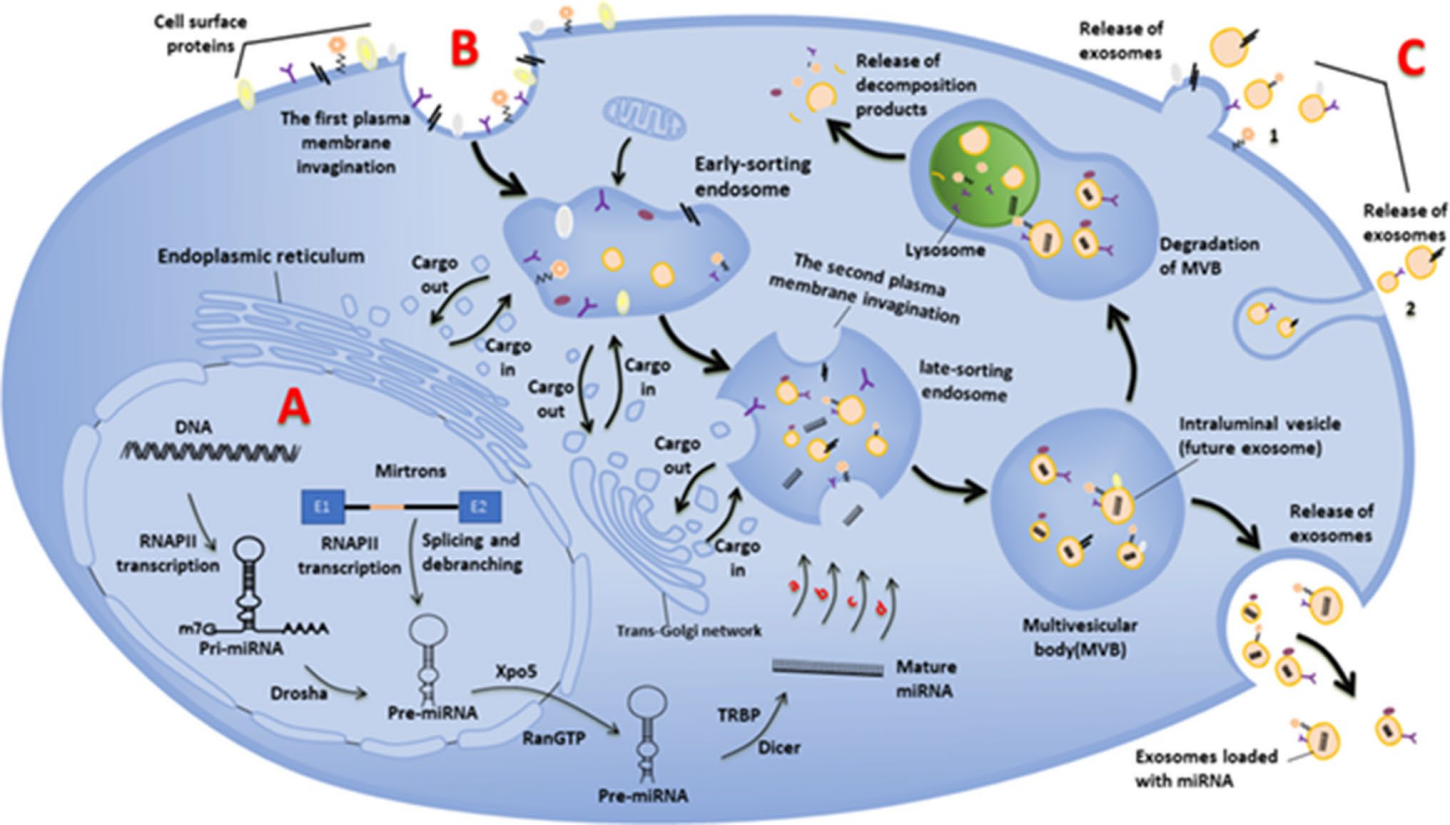
Biogenesis of exosome-loaded miRNAs. **A**: Biogenesis of miRNA, the miRNA selective loading pathway for exosomes: a nSMase2-dependent pathway; b the miRNA motif and sumoylated hnRNPs-dependent pathways; c the 3′-end of the miRNA sequence-dependent pathways; d the miRNA induced silencing complex-related pathways. **B**: Exosomes biogenesis, the two plasma membrane invaginations during exosome formation and release of exosomes. **C**: Other possible models for the biological origin of exosomes: 1 directly released by the budding vesicles of the plasma membrane; 2 It buds in intracellular plasma membrane-connected compartments and subsequently releases exosomes by unblocking the narrow IPMC neck

Almost all types of cells, prokaryotic and eukaryotic, release extracellular vesicles (EVs) as part of their normal physiology and during acquired abnormalities [7]. Although research on EVs is ongoing and classification of EVs continues to advance, they can be broadly separated into two categories, ectosomes and exosomes. Ectosomes are large vesicles with a diameter of 50 nm ~ 1 µm, which sprout directly from the plasma membrane and contain microbubbles and particles [8]. By contrast, exosomes, derived from endosomes and the plasma membrane, range in diameter from 30 to 200 nm. In 1985, it was first reported by B T Pan, K Teng, C Wu, M Adam, R M Johnstone, and others that there were exosomes budding inward through the plasma membrane to form endosomes in the nucleus during the maturation of sheep reticulocytes [9]. In the following decades, researchers have conducted more in-depth research on the biological sources of exosomes. As a subcellular vesicle with a lipid bilayer structure, the biogenesis of exosomes involves two invaginations of the plasma membrane [7]. The first plasma membrane invagination forms the early-sorting endosomes (ESEs), which contain cell surface proteins and soluble proteins associated with the extracellular environment. In addition, the plasma membrane invagination can also directly fuse with the formed ESEs in some cases. The trans-Golgi network and endoplasmic reticulum also contribute to the formation of ESEs [10]. ESEs further mature into late-sorting endosomes (LSEs), in which the second plasma membrane invagination occurs, and when the plasma membrane invagination is completed, the LSEs become multivesicular bodies (MVBs), which contain intraluminal vesicles (future exosomes) [7]. As the plasma membrane invaginates, this leads to the incorporation of cytoplasmic components, resulting in a variety of novel intraluminal vesicles with different roles and functions. MVBs can not only fuse with lysosomes or autophagosomes for degradation, but also with the plasma membrane to release exosomes [11]. However, not all exosomes are derived from endosomes, and there are also exosomes that bud from the plasma membrane [4]. Unfortunately, most scholars did not pay attention to this, so most reference maps do not show exosomes of plasma membrane origin. There are three possible biological origins of exosomes [10]: (1) direct released of the budding vesicles in the plasma membrane, (2) budding from intracellular plasma membrane-connected compartments (IPMCs) and subsequent release of exosomes by unblocking the narrow IPMC neck, and (3) vesicle budding forms discrete endosomes that mature into multivesicular bodies that release exosomes upon fusion with the plasma membrane. In addition, some studies have shown that RAS-related GTPase RAB, syndecan-1, ESCRT, phospholipids, quadriceps protein, ceramide, sphingomyelinase, snare, and Alix (apoptosis-related gene-2-interacting protein X) are involved in the origin and biogenesis of exosomes, but their precise rate-limiting roles and functions in exosome biogenesis in vivo need to be further explored (Fig. 1) [7, 12, 13].

## Exosome Functions

Since E G Trams, C J Lauter, N Salem Jr, and U Heine first found vesicles surrounded by a lipid bilayer in the supernatant of sheep reticulocytes cultured in vitro, that is, exosomes, researchers have been fascinated by this subcellular vesicle with an average diameter of only about 115 nm [14]. The function of exosomes has also been further appreciated in the continuous efforts of researchers. Initially, exosomes were only considered to clean up degraded and unwanted cellular components. However, it is now accepted that exosomes have a role in transmitting cellular signals, i.e., intercellular communication. For example, 40–75-nm brain-derived exosomes deliver pro-survival signals to recipient cells, 75–100-nm pancreas-derived exosomes transmit pro-apoptotic signals to recipient cells, and 100–160-nm liver-derived exosomes deliver pro-survival signals to recipient cells [7]. In addition, exosomes play meaningful roles in mammalian reproductive development, cancer, neurodegeneration, and immunity. Exosomes may also have the potential to diagnose and track diseases, or they may be exclusive biomarkers of diseases. Because exosomes are secreted by almost all cells and can be found in almost all body fluids, including blood, urine, cerebrospinal fluid, breast milk, and amniotic fluid, there is no doubt that exosome-based fluid biopsies can be more easily sampled than traditional tests [15].

## Biogenesis and Loading of miRNAs in Exosomes

It is well known that exosomes play a role in intercellular communication, and this role is related to the contents that exosomes are loaded with, which includes various coding and non-coding RNAs (such as miRNAs), lipids, and proteins [16]. miRNAs have been proposed to be the key to post-transcriptional regulation of gene expression in multicellular organisms and have received much attention as one of the important components of exosomes. The biogenesis of miRNAs includes two stages, the first of which takes part in the nucleus and the other in the cytoplasm. In the first step inside the nucleus, the RNA polymerase II (RNAPII)-specific transcripts of independent genes or of represented introns of protein-coding genes form the major precursor (pri-miRNA), and the pri-miRNA is subsequently processed into a precursor hairpin of about 70 nucleotides (pre-miRNA) with the help of Drosha from the RNase III enzyme family [17]. There is also a small fraction of pre-miRNAs that are not processed by Drosha but are generated from very short introns (mirtrons) by splicing and debranching [17]. The pre-miRNA then enters the cytoplasm with the help of nuclear complexes composed of Xpo5 and RanGTP and is processed into mature miRNA by Dicer from the ribonuclease III family with the help of transactivation-responsive RNA binding protein (TRBP) [18]. Also in mammals, argonaute 2 (AGO2) has a potent RNase-like endonuclease activity and is able to form a processing intermediate called AGO2 cleavage precursor miRNA (ac-pre-miRNA) that supports Dicer processing by cleaving the 3′arm of some pre-miRNAs [19]. In parallel, miRNAs are selectively packaged into exosomes. For example, miR-451 is the most abundant in exosomes of HEK293T cells [20]. Although the mechanism that selects which miRNA to load into exosomes is unclear, there are currently four possible models, including (1) the nSMase2-dependent pathway, (2) the miRNA motif and sumoylated hnRNP-dependent pathway, (3) the 3′-end of the miRNA sequence-dependent pathway, and (4) the miRNA-induced silencing complex (miRISC)-related pathway (Fig. 1) [21].

## Reasons for Using Exosomes as miRNA Carriers in SCI

miRNAs are a class of non-coding RNAs that mediate post-transcriptional gene silencing by binding to the 3′-untranslated region (UTR) or open reading frame (ORF) of the target mRNA. miRNA were shown to be involved in various pathological processes and play an active role in disease development. In SCI, miRNAs inhibit inflammatory factors, reduce axonal apoptosis, inhibit glial cell activation, and promote neuronal repair. In addition to exosomes that use miRNA as cargo, high-density lipoprotein (HDL) and extravesicular argonaute 2 (AGO2) protein play a role in the transport of miRNAs [22, 23]. It was also found that miRNAs are stable in body fluids such as blood, breast milk, saliva, and urine [21]. miRNA loaded onto HDL also play a role in intercellular communication related to lipid metabolism, inflammation, and atherosclerosis, which has been instrumental in the development of therapies for cardiovascular disease (CVD) [22]. Unfortunately, no attention has been paid to the role of HDL-miRNA in SCI. Regarding the role of miRNAs attached to the AGO2 protein in SCI, the team of Dr. Camelia A. Danilov reported that intravenous administration of miRNAs bound to the AGO2 protein improved the recovery of contused spinal cord experimental mice and modified expression of ECM genes in the injured spinal cord reducing fibrotic scar formation, in addition to reducing activated small collagen cell/macrophage numbers to improve spinal cord recovery [24]. Because of their specificity, the study of exosomes has attracted more research enthusiasm than the use of HDL and AGO2 as carriers to transport miRNAs for the treatment of SCI. Exosomes can protect their payload from enzymatic breakdown or other processes because of their lipid bilayer structure, which ensures the safety and stability of the cargo [25]. Exosomes are also able to cross the blood–brain barrier (BBB) [1], where almost 98% of systemic drugs cannot pass. Moreover, exosomes as miRNA carriers can also enable targeted input to recipient cells. For example, it was demonstrated that exosomes specifically deliver miRNAs to epidermal growth factor receptor (EGFR)-expressing breast cancer cells using engineered donor cells expressing a transmembrane structural domain of the platelet-derived growth factor receptor fused to the GE11 peptide receptor, while the team also found that exosomes containing let-7a miRNA were expressed in EGFR-expressing xenograft breast cancer tissue when administered intravenously to RAG2(− / −) experimental mice [26]. The non-immunogenic nature of exosomes allows for greater stability in possible future clinical studies. In addition, in cells that specifically express miRNAs, the exosomes of that cell are more likely to contain that specific miRNA, a property that has a significant positive effect in the extraction of the target exosome miRNA and in the study of what role the exosomes carrying miRNAs play in disease. It was reported that miR-133 expression was significantly elevated in exosomes secreted by adipose-derived stem cells (ADSCs) under miR-133b modification [27]. All of the above characteristics suggest that exosomes may be ideal miRNA carriers for SCI treatment.

## Exosomes as miRNA Carriers for Exploring Potential Mechanisms of SCI

The complex pathophysiological process of SCI includes the initial stage, which involves neuronal death, blood–brain barrier damage, and vascular rupture, as well as the second stage of injury, which involves neurogenic apoptosis, inflammatory response, vascular remodeling, glial cell activation, and other secondary aggravating processes.

**Fig. 2:**
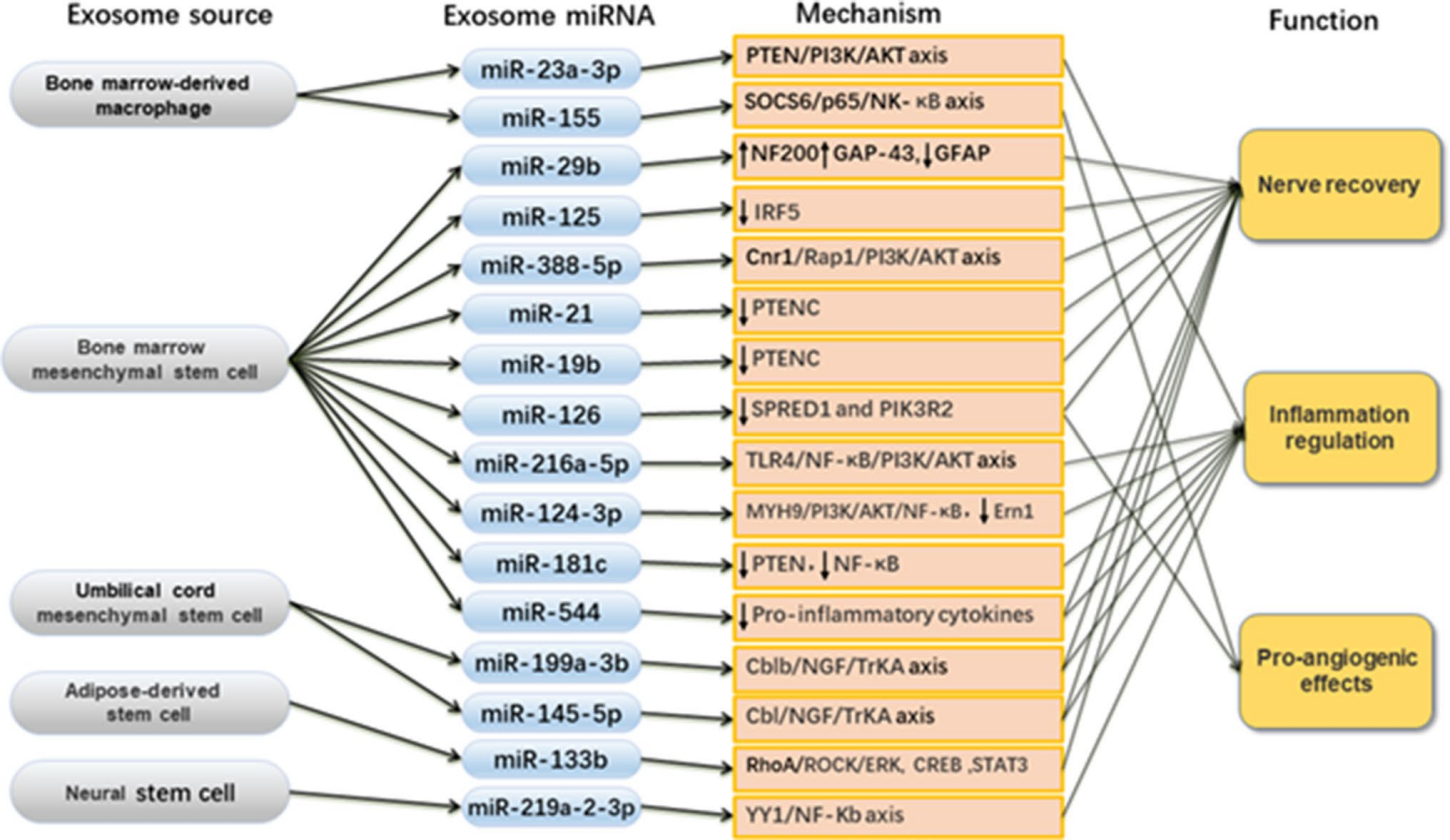
Cellular origins, types, mechanisms, and functions of exosome-loaded miRNAs

However, little is known about the mechanisms that cause these changes and what pathways can promote or inhibit them. Because SCI is a complex pathological process, it is important to explore the underlying mechanisms not only to uncover the relevant mechanisms and pathways, but also to provide guidelines for future clinical applications. Because of the unique role of exosomes in intercellular communication and because miRNA is an important part of exosomes, there is a place for exosomes as miRNA carriers to explore the potential mechanisms of SCI. A recent study used a spinal cord contusion mouse model to study the effect of macrophage infiltration on vascular changes after SCI [28]. Primary bone marrow-derived macrophages (BMDM) from the tibia and femur of 4-week-old mice were used to culture M1-polarized macrophages as source of miR-155 exosomes. The exosome-loaded miR-155, which accumulates in M1-polarized macrophages at the blood-spinal cord barrier (BSCB) after SCI, internalizes miR-155 targets in vascular endothelial cells and reduces the expression of SOCS6, which ubiquitinates and degrades p65 in the cytoplasm [28]. Due to of SOCS6 inhibition, proteasomal degradation of p65 is inhibited, its nuclear translocation is upregulated, and it subsequently activates the NK-kB pathway, which in turn promotes EndoMT and ROS production in vascular endothelial cells, impairs mitochondrial function, and disrupts BSCB integrity. The results of the said study may enhance our understanding of the relationship between macrophages and vascular endothelial cells and reveal potential mechanisms regulating BSCB integrity after SCI [28]. In addition to this research, stem cells are also used as experimental tools to generate miRNA-loaded exosomes, mostly using bone marrow mesenchymal stem cells (BMMSCs). And it has been reported that MSC-derived EVs exert most of the positive effects of direct MSC use as a treatment for nerve disorders, but without the risks associated with stem cell transplantation [29]. However, umbilical cord MSCs, adipose-derived stem cells, and neural stem cells have also been used as vehicles for exosome generation (Fig. 2). In addition, most studies related to stem cell-derived exosome-loaded miRNAs used SD rats as experimental models, but only miR-124-3p-related studies have recorded the use of mice as animal experimental models [32], and the SCI models were made by dropping iron bars at a certain height to reach.

### Exosomal miRNAs Secreted by BMMSCs Used to Explore the Mechanisms of SCI

In most of the studies using bone marrow MSCs for exosome extraction and isolation, several miRNAs have been identified that play an active role in SCI and are considered to have potential in SCI therapy, and the mechanisms of these miRNAs have also been investigated.

#### miR-125 and miR-124-3p

Both these miRNAs play roles in regulating M2 macrophages, which not only key effector cells in the inflammatory response to SCI, but the repair of SCI is also based on macrophage activation. miR-125 promotes M2 macrophage polarization as an upstream factor and improves SCI by negatively regulating IRF5, while miR-124-3p promotes M2 macrophage polarization by negatively regulating Ern1 [30, 31]. It was also found that miR-124-3p inhibits the activation of M1 microglia and microglia-induced neuroinflammatory responses through the MYH9/PI3K/AKT/NF-κB signaling pathway and thereby inhibits A1 astrocytes [32].

#### miR-21 and miR-19b

These miRNAs are found in exosomes derived from bone marrow mesenchymal stem cells, which inhibit neuronal apoptosis and promote neuronal differentiation in SCI by a similar mechanism. When their abundance in exosomes is increased, they inhibit phosphatase and tensin homologs (PTENC), which in turn inhibit neuronal apoptosis and promote neuronal differentiation [33–35].

#### miR-216a-5p and miR-181c

In addition, both miR-216a-5p and miR-181c have regulatory effects on the activation of NF-κB signaling. miR-216a-5p influences the activation of NF-κB signaling by regulating the downstream target gene TLR4. miR-216a-5p inhibits the activation of NF-κB signaling by suppressing the target gene TLR4, which subsequently activates PI3K and AKT. As a result, the TLR4/NF-κB/PI3K/AKT signaling cascade is activated and then promotes the conversion of microglia from the pro-inflammatory M1 to the anti-inflammatory M2 phenotype, thus playing a role in the repair of traumatic SCI. By contrast, miR-181c inhibits microglia inflammation by inhibiting PTEN, and also inhibits NF-κB activation to alleviate inflammation and apoptosis caused by SCI [36, 37].

#### miR-388-5p and miR-126

Bone marrow MSC-derived exosome-loaded miR-388-5p increases cAMP accumulation through miR-338-5p by downregulating the expression of the target cannabinoid receptor 1 gene (Cnr1), which then activates Rap1, which in turn activates the PI3K/AKT pathway to inhibit apoptosis and enhance neuronal survival. Similarly, marrow MSC-derived exosome-loaded miR-126 promotes angiogenic migration by inhibiting the expression of SPRED1 and PIK3R2 to promote SCI recovery [38, 39].

### Exosomal miRNAs Secreted by Other MSCs Used to Explore the Mechanisms of SCI

In addition to the above studies of bone marrow MSC-derived exosome-loaded miRNAs, there are also studies of exosome-loaded miRNAs from umbilical cord MSCs, such as miR-199a-3p and miR-145-5p, as well as adipose stem cell-derived miR-133b and neural stem cell-derived miR-219a-2-3p.

#### miR-199a-3p and miR-145-5p

There are several potential mechanisms through which miR-199a-3b and miR-145-5p carried by umbilical cord MSC-derived exosomes may contribute to the repair of SCI. miR-199a-3p acts on the direct target cblb, and miR-145-5p acts on the direct target cbl, both of which have inhibitory effects on their direct targets. The resulting inhibition of cblb and cbl downregulates TrKA ubiquitination, which in turn activates the NGF/TrKA pathway to promote AKT and Erk activation, thereby reducing inflammation levels after SCI [40, 41].

#### miR-133b and miR-219a-2-sp

RhoA is a direct target of miR-133b, which is found in exosomes of adipose-derived stem cells. miR-133b downregulates RhoA, which in turn regulates Rho-related kinase (ROCK) to promote ERK phosphorylation, which protects neurons from apoptosis. In addition, miR-133b also promotes CREB and STAT3 phosphorylation, thereby promoting axonal regeneration after SCI [42]. The neural stem cell-derived miR-219a-2-sp achieves inflammatory suppression by downregulating the YY1 gene and thereby inhibiting the NF-κB pathway [43].

Although there is some understanding of the potential effects of exosomal miRNAs on SCI, the current knowledge is limited, and it is worth exploring whether there is a common target receptor for miRNAs, whether there is a common molecular signaling pathway, and what effects mixed miRNAs might have on SCI. In addition, almost all studies have used rodents in the selection of animal models of SCI. It is hoped that the range of experimental animals can be expanded to include animals with more complex spinal cord structures (e.g., dogs) to explore the underlying mechanisms of SCI.

## The Role of Exosome-loaded miRNAs in the Pathophysiology of SCI

SCI is divided into primary and secondary injuries. Primary injuries occur when the spine is suddenly injured, resulting in fractures and vertebral dislocations and therefore carrying the hallmarks of bone fragments and spinal ligament tearing, and include destruction of the neural parenchyma, disruption of the axonal network, hemorrhage, and glial membrane disruption [44]. The primary determinants of spinal cord injury severity are the degree of initial damage and the duration of spinal cord compression. The sequence of events associated with secondary injury is activated by the onset of biochemical, mechanical, and physiological changes within the neural tissue. Secondary injury is divided into three stages: acute, subacute, and chronic injury. After the primary injury stage, the onset of the acute secondary injury stage is characterized by clinical features such as vascular damage, ionic imbalance, excitotoxicity, free radical production, increased calcium inward flow, lipid peroxidation, inflammation, edema, and necrosis [45]. If the acute secondary injury phase persists, the subacute secondary injury phase begins and is characterized by neuronal apoptosis, axonal demyelination, walleye degeneration, axonal remodeling, and glial scar formation [45]. Subacute secondary injury leads to a chronic secondary injury phase of SCI characterized by formation of cystic cavities, axonal necrosis, and glial scar maturation [46]. Recent studies have shown that exosome-loaded miRNAs can aid the recovery of SCI in experimental mice, including the ability to promote neuronal recovery, inhibit apoptosis, promote axonal regeneration, modulate the inflammatory response, and promote angiogenesis, as well as to regulate microglia and macrophages. The available studies on the effects of exosome-loaded miRNAs on SCI can be broadly classified into three categories, including (1) neurological recovery: neuronal regeneration, apoptosis inhibition, and axonal regeneration; (2) inflammatory response regulation: microglia, astrocytes, and macrophages regulate and downregulate pro-inflammatory factors; and finally (3) pro-angiogenic effects (Fig. 2). This demonstrates the potential of exosome-loaded miRNAs as a tool for SCI treatment.

### The Role of Nerve Recovery in SCI

In terms of neurorestorative effects, a number of exosome-loaded miRNAs have been demonstrated to play a positive role in animal studies and portend future potential for clinical therapeutic use, including miR-29b, miR-199a-3p, miR-145-5p, miR-21, miR-19b, miR-338-5p, miR-133b, and miR-544.

#### Neuronal Regeneration

In terms of neuronal regeneration in neurorestorative effects, it was reported that injection of miRNA-29b exosomes significantly increased the number of neurofilament protein 200 (NF200)- and growth-associated protein-43 (GAP-43)-positive neurons while decreasing the number of glial fibrillary acidic protein (GFAP)-positive neurons in experimental rats with SCI [47]. NF200 is a structural protein of nerve cells and axons [48]. GAP-43 is a specific phosphoprotein on vertebrate nerve cell membranes and is considered to be a marker of synaptic plasticity, neuronal development, and regeneration [49]. GFAP is a cytoskeletal protein and a major component of glial cells. Early in SCI, GFAP expression is upregulated and promotes axonal regeneration, but late in SCI, the inhibition of axonal growth by massively proliferating glial scar tissue is dominant compared to the role of GFAP in promoting axonal regeneration [50]. Wang et al. used umbilical cord MSC-derived exosomes to discover that miR-199a-3p/145-5p may promote recovery of motor function in rats with SCI by targeting the Cblb and Cbl genes to regulate the flipping and/or activation of TrkA, which is indispensable in the developmental maturation of the nervous system [40, 41].

#### Apoptosis Inhibition and Axonal Regeneration

miR-338-5p overexpressed in MSC-derived exosomes was found to reduce apoptosis and promote neuronal survival through the Cnr1/Rap1/Akt pathway [39]. In addition, exosomes carrying miR-21 and miR-19b isolated from differentiated PC12 cells and MSCs exhibited a protective effect in SCI treatment by suppressing PTEN expression while also regulating apoptosis and differentiation of neuronal cells in SCI patients [34, 35, 51]. PTEN protein, a tumor suppressor encoded by a gene on chromosome 10q23.31, can regulate many signaling pathways through phosphatase-dependent and phosphatase-independent mechanisms [52]. In addition, it was reported that when miR-133b exosomes were injected intravenously into the tail vein of rats, the treatment protected neurons, promoted axonal regeneration, and improved the recovery of motor function in the hind limbs after SCI [53]. It was also reported that miR-126 exosomes from mesenchymal stem cell-filled cells promote angiogenesis and neurogenesis, inhibit apoptosis, and promote functional recovery after SCI [38]. In addition, miR-544 from bone marrow mesenchymal stem cells was found to not only reduce the number of apoptotic neurons, but also significantly inhibit the production of pro-inflammatory cytokines after SCI [54]. Furthermore, miR-125a was found to not only promote M2 macrophage polarization, but also inhibit neuronal apoptosis and improve SCI recovery [31]. It was also found that miR-494 contained in exosomes from murine mesenchymal stem cells not only inhibits apoptosis and promotes neurofilament regeneration, but also suppresses inflammatory responses and regulates various inflammatory factors [55]. This indicates that there may be multiple protective mechanisms of miRNAs in SCI.

### The Role of Inflammation Regulation in SCI

The inflammatory response plays a crucial role in the development of SCI, and excessive inflammation may cause severe damage [56]. The regulation of the inflammatory response in SCI includes the regulation of inflammation-associated cells such as microglia, astrocytes, and macrophages, as well as the regulation of pro-inflammatory factors.

#### Inflammatory Regulation by Microglia, Astrocytes, and Macrophages

Microglia are the resident macrophages of the central nervous system (CNS). Following SCI, microglia play an important role in the activation and modulation of neuroinflammation. Microglia can be divided into M1 and M2 phenotypes according to their functional roles in the inflammatory response. M1 microglia are considered to be pro-inflammatory/induce injury [32], while M2 microglia are thought to have an anti-inflammatory effect [36], so the search for miRNAs that promotes the polarization of microglia toward the M2 anti-inflammatory phenotype is of great significance for the treatment of SCI. There is further evidence that macrophages have both a pro-inflammatory, cytotoxic M1 phenotype and an anti-inflammatory, pro-repair M2 polarization state [57]. Studies to date have shown that exosome-loaded miR-216a-5p, miR-124-3p, and miR-181c regulate microglial polarity; miR-181c attenuates microglial inflammatory responses; and miR-216a-5p shifts microglia from an M1 pro-inflammatory phenotype toward an M2 anti-inflammatory phenotype, while miR-124-3p inhibits the activation of M1 microglia and A1 astrocytes [30, 36, 37].

Moreover, miR-124-3p has a regulatory effect on macrophages in addition to microglia and can also ameliorate neurological damage caused by SCI by inhibiting Ern1 expression and enhancing M2 polarization [31]. In addition, miR-145-3p plays a negative regulatory role in the regulation of astrocyte proliferation downregulating smad3 protein activity [58]. It was also shown that bone marrow-derived, exosomal microRNA-125a can promote M2 macrophage polarization in SCI by downregulating IRF5 [31]. In addition to what was mentioned above, a research team found that M2 macrophage-derived exosomes can increase the percentage of M2 macrophages and decrease the percentage of M1 macrophages at the site of injury, while M1 macrophage-derived exosomes produce analogous effects. This promising observation may be associated with miR-23a-3p carried by exosomes produced by macrophages and plays an important role in regulating macrophage polarization through the miR-23a-3p/PTEN/PI3K/AKT axis [59].

#### Downregulation of Pro-inflammatory Factors

There are also other miRNAs that promote SCI recovery by regulating inflammation levels. For example, miR-219a-2-3p inhibits neuroinflammation, suppresses apoptosis, and promotes neural regeneration after SCI, miR-199a-3p/145-5p reduces inflammation levels by regulating AKT and ERK activation, while miR-544 inhibits the production of the pro-inflammatory cytokines IL-1a, TNF-a, IL-17B, and IL-36bin following SCI [41, 43, 54].

### The Role of Angiogenesis in SCI

Pro-angiogenic effects also play a role in recovery from SCI by playing an active part in maintaining barrier integrity. There is evidence that when mitochondrial function of vascular endothelial cells is impaired in cerebrovascular disease, this in turn impairs the integrity of the blood–brain barrier [60]. It was also found that when miR-155 carried by exosomes from M1-polarized macrophages was downregulated, it reduced vascular endothelial cell damage and subsequently protected the integrity of the blood-spinal cord barrier (BSCB) [28]. The rupture of BBB or BSCB after SCI usually leads to edema, upregulation of the inflammatory response, progressive neuronal death, and glial cell activation, so the role of angiogenesis promotion in maintaining the integrity of the blood–brain barrier and blood-spinal cord barrier for promoting recovery from SCI becomes apparent [46]. In addition to the finding that miR-155 in exosomes derived from M1-polarized macrophages promotes EndoMT and impairs mitochondrial function by activating the NF-κB signaling pathway in vascular endothelial cells, it was also reported that miR-126 promotes angiogenesis and neurogenesis while also reducing apoptosis in rats after SCI, as well as improving the recovery from SCI in rat [38]. Unfortunately, studies on the use of exosome-loaded miRNAs to promote angiogenesis to improve recovery from SCI have not attracted the same attention that was given to studies on modulating the inflammatory response and improving neurological recovery. The significance of promoting angiogenesis to improve the recovery from SCI should therefore be more thoroughly explored in the future.

SCI pathophysiology is complex and currently in the exploratory stage. The positive effects of exosome-loaded miRNAs on neural recovery, inflammatory response modulation, and pro-angiogenesis during the acute and subacute phases of SCI suggest the possibility of using exosome-loaded miRNAs in SCI treatment. However, the role of exosome-loaded miRNAs in the chronic phase of SCI scarring is not known. However, it has been shown that exosome-loaded miR-29a from modified adipose-derived mesenchymal stem cells reduces excessive scar (mainly hypertrophic scars and keloids) formation by inhibiting TGF-β2/Smad3 signaling Therefore, there may be an exosome-loaded miRNA that can improve the SCI chronic phase scarring.

## Perspectives

### Exploring the Synergistic Effects of Multiple Types of Exosome-Loaded miRNAs

In studies on the use of exosome-loaded miRNA for SCI treatment, there is a lack of research on the synergistic effects of multiple types of exosome-loaded miRNAs in SCI. However, the question of whether there is a synergistic effect of multiple species of miRNA in disease progression has actually been investigated to some extent. For example, in a study on osteoporosis, it was found that miR-708-5p inhibited osteoclast differentiation by targeting SMURF2, while miR-708-3p promoted osteoclast differentiation by regulating the expression of CDR1as. This suggests that miR-708-5p and miR-708-3p affect osteoclast differentiation through different mechanisms and exert synergistic effects in the development of osteoporosis through different avenues [61]. In addition, it was found that miR-129 and miR-335 promoted diabetic wound healing by inhibiting Sp1-mediated MMP-9 expression [62]. In glioma, miR-200c and miR-141 were found to synergistically suppress ZEB1 to inhibit glioma cell growth and migration [63]. In studies of exosome-loaded miRNAs for the treatment of SCI, we have found many miRNAs with the same therapeutic effects but have seen few reports of studies combining miRNAs with similar therapeutic effects to analyze whether there are synergistic effects, including for example the presence of miR-21 and miR-19b in bone marrow mesenchymal stem cell exosomes that both inhibit neuronal apoptosis and promote neuronal differentiation. When the abundance of these miRNAs in exosomes is upregulated, they inhibit phosphatase and tensin homolog (PTENC) proteins, thus acting as inhibitors of neuronal apoptosis [33–35]. This research direction on the synergistic effects of multiple species of miRNAs in SCI provides new ideas for understanding the mechanism of SCI and the benefits of exosomes and exosome-loaded miRNAs in SCI, as well as for studying possible future treatments for SCI.

### Exploring the Synergistic Effect of Exosome-Loaded miRNAs with Hydrogels for SCI Treatment

Although numerous studies have shown that exosome-loaded miRNAs can effectively alleviate damage caused by SCI and improve the healing effect, the therapeutic effect of exosome-loaded miRNAs is also related to the half-life and clearance rate of exosomes in vivo. According to a previous study, exosomes secreted by B16BL6 melanoma cells were rapidly cleared from the circulation of mice, with a half-life of about 2 min after injection [64]. Therefore, it is crucial to increase the half-life of exosomes in vivo to improve their therapeutic efficacy. Hydrogels are physically or chemically cross-linked three-dimensional hydrophilic polymer networks that can adsorb large amounts of water in aqueous solutions without undergoing a dissolution process [65]. Studies have shown that cells or bioactive molecules retain their structure and function for longer periods of time when incorporated into a hydrogel [66]. Thus, hydrogels may be an ideal substance to encapsulate exosomes loaded with miRNAs, increasing the half-life and decreasing the clearance rate, thus improving the curative effect. Recently, a research team has genetically engineered synovial-derived MSCs to overexpress miRNA-126-3p and mixed them with chitosan biopolymers to form hydrogel-exosome complexes that contain large amounts of miRNA-126-3p and release exosomes in a controlled manner [67]. A research team also isolated miRNA-675-containing exosomes from supernatants of human umbilical cord MSCs and mixed them with sonicated filipin solution to form miRNA-675 exosome-filipin hydrogels. The filipin hydrogels consistently released exosomes under in vitro and in vivo conditions, while increasing exosome retention and miRNA-675 half-life [68]. In another study, researchers found that exosomes from adipose-derived mesenchymal stem cells could be effectively incorporated into FHE hydrogels with a durable sustained release period. Compared to the free exosome control group, the FHE hydrogel exosomes continued to promote wound healing, collagen deposition, and hair growth in a mouse diabetic wound model at postoperative day 21 [69]. This suggests that the incorporation of exosome-loaded miRNAs into hydrogels offers the opportunity to extend their half-life and improve the therapeutic efficacy. Therefore, the application of hydrogel-encapsulated exosomes loaded with miRNAs in SCI has significant potential and may become a future research direction for SCI treatment.

### Challenges of Exosome-Loaded miRNAs for SCI Treatment

Exosome-loaded miRNAs have tremendous potential clinical application as a therapeutic tool for SCI. First, although many studies have shown a positive effect of exosome-loaded miRNAs on SCI recovery, most of them are rodent-based, especially Sprague Dawley rats (SD rats), and a few are mice [28, 32], and there are anatomical differences between human and rodent spinal cords. Rodent models have a smaller SCI area, while humans typically have a larger SCI area, which results in more tissue loss. In addition, the human nervous system is more complex and advanced than the rodent nervous system. Therefore, further experiments should be expanded to use animals that are larger and have more complex and advanced nervous systems (e.g., dogs) for further studies [70]. In addition, the potential risks of exosome-loaded miRNAs therapy for SCI should be fully understood before clinical SCI treatment. miRNAs may have the ability to act on multiple targets, for example, miR-214 can mediate cardiac fibroblast proliferation and collagen synthesis not only by inhibiting Mfn2 and activating ERK1/2 MAPK signaling, but also by inhibiting TGF-β1 and MMP-1/TIMP-1 regulation prevents fibrosis and exerts cardioprotective effects [71, 72]. Therefore, the use of miRNAs for disease treatment may adversely affect other fundamental biological pathways. Therefore, a full understanding of the risks seems essential. However, the current studies seem to focus more on the positive effects of exosome-loaded miRNAs for SCI treatment without noting the potential risks that may arise, which limits the use of exosome-loaded miRNA for clinical SCI treatment. There are still technical challenges in exosome production. There is no consensus on the method of exosome isolation. Different methods of exosome isolation have their own advantages and disadvantages. In fact, there are significant differences in protein and RNA content between isolation methods. The most commonly used method is ultracentrifugation, but the purity of exosomes obtained by this method is low, and exosomes are contaminated with other EVs of similar diameter [70], so it is necessary to explore a more efficient and uniform approach. Further research is also still needed for exosome storage, preservation, and transport, and a study published in 2018 showed that further purification and improved storage, preservation, and transport of exosomes could be achieved by lyophilization [73]. However, further studies are needed to demonstrate whether lyophilization alters the properties of exosomes. Therefore, we need to develop more advanced technologies for exosome isolation, extraction, detection, preservation, and transport to further our understanding of exosome-loaded miRNAs as a way to overcome the challenges.

### Future Directions of Exosome-Loaded miRNAs for SCI Treatment

With the progress of research on exosome-loaded miRNAs for SCI treatment, studies revealed that exosome-loaded miRNAs play various roles in modulating aspects of SCI, including neurological recovery, inflammatory regulation, and pro-angiogenic effects. Future research on exosome-loaded miRNAs may be focused on exploring the synergistic effects of multiple types of miRNAs in SCI or exploring the synergistic effects of exosome-loaded miRNAs embedded in a hydrogel.

## Conclusions

Exosomes are membrane-bound vesicles with a diameter of only 30–200 nm that can be secreted by almost all cells with important roles in disease development, disease treatment methods, and studies on the potential mechanisms of disease. In the exploration of exosomes for SCI, they are not only a tool to explore potential mechanisms of SCI, but may be the key to cure SCI in the future. The miRNAs contained in exosomes may be the key to unlocking the cure for spinal cord problems. Experimental animal models showed that exosome-loaded miRNAs had a significant therapeutic effects in mice with SCI. The exosome-loaded miRNAs have the effect of improving neural recovery, modulating the inflammatory response, promoting vascular recovery, and improving motor recovery after injury in mice, suggesting the significance of exosome-loaded miRNAs in SCI. Regardless of the fact that good results were obtained in experimental animals, there is still an urgent need for clinical trial results to further verify whether exosome-loaded miRNAs have a positive effect on SCI. Meanwhile, in terms of the clinical importance and significance of exosome-loaded miRNAs for the treatment of SCI, it is regrettable that most of the innovations for exosome-loaded miRNAs are only used in experimental animals, and there are few experiments for clinical use, and there are still many difficulties for clinical use, such as the effective production rate of stem cells for exosome-loaded miRNAs, so it is difficult to achieve the summary for clinical use, but it can be seen from the relevant animal experiments the future of exosome-loaded miRNAs in clinical SCI. It is expected to be a powerful force in clinical SCI. Therefore, we need to investigate how to improve the efficiency of exosome-loaded miRNA delivery to the recipient cells, how to prolong the therapeutic effect of exosomes, and how to simplify the isolation, extraction, and detection of exosomes. In conclusion, the application of exosomes loaded with miRNA sin SCI is a promising research and application direction because exosomes can effectively cross the blood–brain barrier and can be used for the treatment and diagnosis of many central nervous system diseases including SCI. Improving the stability, targeting and safety of exosomes loaded with miRNAs will be important issues in the treatment of SCI in the future.

## Data Availability

Not applicable for that section.
